# Histories of sardine fisheries. architecture and environment in the Bay of Biscay (1846–1965)

**DOI:** 10.12688/openreseurope.23094.2

**Published:** 2026-07-14

**Authors:** Alice Nouvet, André Tavares, Giulia Secci-Petretto, Rafael Sousa Santos, Garðar Eyjólfsson, Sónia Gabriel, Claudia Soares, Diego Inglez de Souza, Neftalí Sillero

**Affiliations:** 1 Universidade do Porto Faculdade de Arquitectura, Porto, Porto District, Portugal

**Keywords:** Fishing architecture, Fish landing records, Historical fisheries, Multiscalar research, Sardine, Statistical analysis (Granger Causality Test, Generalized Linear Model), Interdisciplinary research

## Abstract

**Background:**

This study combines historical cartography and statistical analysis to examine France Atlantic’s sardine fisheries across time (1846–1965) and spatial scales, from individual harbours to the continental shelf. It aims to use interdisciplinary methods to identify the relations between European sardine populations ( *Sardina pilchardus*), climatic fluctuations, and the material structures associated with fishing—including vessels and coastal infrastructures. The underlying hypothesis is that long-term sardines fluctuations in landings influenced the way we built on land and, in turn, exerted pressures on sardines ecologies.

**Methods:**

Historical data on fishing vessels, coastal infrastructures, sardine landings, territorial occupation by canneries, fishing grounds, and fisher censuses were compiled to reconstruct the temporalities of fishing landscapes. First, we quantified the spatial expansion of canneries and shoreline infrastructures. While canneries were the product of private industrial investment, shoreline infrastructures included both private and public developments. Given the limited availability of detailed funding records, subsequent analyses focused on publicly funded coastal works, for which investment amounts and construction timelines could be reconstructed and compared with profits derived from fish landings. Second, in the absence of consistent financial records or urban plans at the continental-shelf scale, population census data were used as a proxy for urban growth and related to fishing effort to identify areas of intensified pressure. These patterns were then compared with the spatial distribution of fishing grounds. Finally, sardine landings were analysed against environmental indices and vessel characteristics to assess their relative influence.

**Results:**

The assessment revealed: (1) a long-term decline in sardine landings that does not seem to be influenced by environmental variability; (2) the continuing expansion of harbour infrastructures and vessels tonnage even when landing values declined; (3) trends toward the spatial concentration of fishing effort.

**Conclusions:**

The findings suggest that harbour works were driven less by an objective awareness of ecological pressure than by latent development projects activated during fishing crises. Rather than serving as a passive backdrop to the history of fisheries, these infrastructures actively shaped the circulation of sardine landings, capital investment, and fishing activity along the coast.

## Introduction

The historical relationship between fisheries and fish, including its impact on fish populations, has been the subject of research ranging from biology to oceanography ( Pauly, 2019; Jackson et al., 2011). Yet such history has overlooked the connection between the fisheries and the associated dynamics on land. This paper addresses this gap by assessing the architectural and urban dynamics related to historical sardine fisheries in South Brittany, France. Sardine fisheries in the Bay of Biscay became one of the main sources of economic income for the French regions of the Atlantic coast at the turn of the twentieth century ( Fichou, 2006; Binet, 1999). The activities helped shape not only the regional economies but also the shoreline, generating a variety of constructions that defined a specific landscape. However, understanding the historical exchanges between sardine populations and coastal societies requires an approach that addresses sardine fluctuations, fishing practices, and the structures that channelled fishing efforts all at the same time. In the Bay of Biscay, several studies ( Binet 1986, 1999; Planque et al., 2004; Bellier et al., 2007) have explored the interplay between fish population dynamics and environmental fluctuations; however, the fishing pressure is frequently treated as secondary and is difficult to quantify in historical assessments. Le Floc’h’s et al. (2023) work is one of the rare studies that engages directly with technical changes over the course of a century (1900–2017), tracing vessel size, gear shifts, and fleet structure. Although he mentions fishing pressure, he does not measure or quantify its effects. A more recent study by Lahellec et al. (2024) complements this by focusing on how pelagic fleets adapted between 2010 and 2018, combining landings, vessels, and fishers’ knowledge. Both works highlight how regulations alongside economic and technological factors shape fishing effort.

In parallel, historical studies by Binet (1986, 1999) have sought to relate fluctuations in sardine landings to broader climatic and oceanographic changes. Binet’s work used coastal archival data, such as harbour statistics, sailors’ registers, and qualitative descriptions of annual yield (such as “good” or “poor” years). It correlates these materials with reconstructed temperature indices, like historical sea surface temperature.

A more recent body of work ( Planque et al., 2007; Bellier et al., 2007) has focused on the ecological factors, mostly hydrography, to show how these factors influence sardines’ spawning behaviours and distributions. These studies tend to examine ecological patterns in isolation from the industry that harvests them. Planque et al. (2004) developed the concept of “hydrological landscapes”, identifying river plumes and oceanographic currents, such as the upwelling, as shapers of marine habitats where pelagic species may spawn and feed. A complementary study, by Bellier et al. (2007), uses ichthyoplankton surveys to demonstrate how spawning locations have shifted significantly between the late 1960s and early 2000s, likely in response to changing temperatures and salinity.

Building from this understanding of oceanographic structures, a growing body of ecological modelling work has quantified the spatial distribution of sardines in relation to environmental variables. Ecological niche models ( Sillero et al., 2021) have been used to estimate potential spawning and feeding grounds by integrating sea surface temperature, salinity, chlorophyll concentrations, and other hydrographic indicators. For instance, Planque et al. (2007) has identified key thresholds of environmental conditions that influence sardine spawning habitat. The authors divided spawning habitat into three categories: “potential spawning habitat”, which is defined by the hydrographic conditions suitable for spawning; “realized spawning habitat”, the habitat where spawning actually occurs; and “successful spawning habitat”, the habitat where recruitment has happened. Moreover, Hébert-Burggraeve (2023) and Quemper (2021) explored the integration of both commercial and scientific datasets into ecological models that estimate the spatio-temporal variations of small pelagic fish distribution. These modelling approaches not only deepen ecological understanding but also serve as strategic tools for protecting, or rather exploiting more efficiently, sardine populations in changing environmental conditions.

From technical changes to climatic correlations and ecological modelling, these approaches seemingly omit to address the role of built infrastructures in the history of fisheries. Hence, our hypothesis suggests looking at sardine fisheries as a shaper of both the marine and urban ecologies. Indeed, an intertwined process occurred in which the fisheries’ infrastructures developed in response to sardines’ biological traits, while in turn exerting new pressures on the fish and its habitat. It appeared to us that those structures—such as piers, auction houses, and canneries—have been looked at as a passive backdrop, where the fish is accessed, processed, and commodified. By adding the built environment as a layer, our approach aims to reframe the story of the fishing harbours, making architecture an active agent.

To a certain extent, we can argue that shore-based structures are a proxy measurement of fishing pressure, the latter increasing in relation to the growth of fish-processing facilities. In this paper, we explore the hypothesis that fluctuations in sardine landings influenced the ways we built on land. We discovered that this was not necessarily the case, since design and building practices relate to the agendas of a variety of agents other than fish, notably the state (in France through the *Ponts et Chaussées* technical offices). Nonetheless, we can identify a relationship between investment in construction works and reductions in landings, with an influx of public investment to cope with the decrease in fisheries profit. While landing values declined (and we try to establish the reason for this decline, be it environmental factors, overfishing, or circumstantial movements of fish populations, etc.), harbour infrastructures continued to expand. Rather than reacting to an objective awareness of the ecological and environmental factors that might have generated a decrease in landings, the design and construction of terrestrial infrastructure was driven by latent projects, previously unrealized projects whose construction was determined by the urgent need to respond to fishing crises. To address this tangle of reasons, factors, and evidence, this paper explores novel interdisciplinary methods, using the Bay of Biscay historical sardine fisheries as a testing ground.

## Objectives

In this study, our objectives are threefold: firstly, to compile a dataset covering annual sardine landings, fishing effort, and environmental variables (1865–1965)—this dataset served as a basis for assessing fisheries’ economics, climatic fluctuations, and urban transformations across multiple temporalities and scales; secondly, to analyse how the commodification of European sardines and how this process was shaped by the urban patterns and territorial occupation of coastal towns; and thirdly, to examine how the built environment, understood as part of the broader material infrastructure of the sardine fishery, may have reinforced fishing capacity and contributed to pressures on sardine populations.

Our historical data limit our ability to quantify the ecological impacts and to reconstruct the exhaustive material infrastructures of the sardine fishery. Beyond coastal works, canneries, and fishing vessels, sardine production depended on a wider network of shipyards, salt depots, net-drying racks, auction houses, and other facilities for which consistent historical records are lacking. Consequently, our analysis focuses on those dimensions of the fishery for which systematic spatial, temporal, and statistical data were available, including harbour works, canning facilities, vessel tonnage, and census-based indicators of fishing activity. Although these sources provide only a partial reconstruction of the fishery’s material infrastructures, they allow us to examine how investments in harbour facilities and fishing capacity evolved in relation to fluctuations in sardine landings.

While comparing harbour construction work investment with the economic value generated by sardine landings, we observed a recurrent mismatch: even if landing values declined, harbour infrastructures continued to expand. This prompted us to examine the role of the *Corps des Ingénieurs des Ponts et Chaussées*, the state engineering body responsible for harbour works. Their planning practices (phased, anticipatory, and responsive to administrative constraints) help explain why architectural development responded indirectly to sardine fluctuations.

## Methods and materials

### 1. Study area

Located along the Atlantic coast of France and northern Spain, the Bay of Biscay encompasses a continental shelf of approximately 220 000 km ^2^. Its morphology is characterized by a broad northern section (the *plateau armoricain*) that extends nearly 200 kilometres, and a tapering southern section (the *plateau aquitain*) that narrows to about 30 kilometres ( Dessier, 2015). These contrasting shelf widths influence the circulation of water masses and nutrients, resulting in distinct ecological subregions ( Planque et al., 2004). The Bay’s hydrology is defined by the confluence of nutrient-rich river discharges with major oceanic currents. This combination creates a productive coastal ecosystem that supports small pelagic species such as *Sardina pilchardus* (European sardines).

Within this broader context, the study encompasses three scales. The harbour of Douarnenez serves as the local case study, where the most detailed reconstruction of harbour infrastructure was possible, while South Brittany provides a regional framework for examining fishing grounds and changes in vessel tonnage. The broader French Atlantic façade provides the largest geographical frame of analysis. These scales reflect the spatial extent of the historical sources available, and the different dimension of the sardine fishery examined in this study. Across all three scales, sardine landings data serve as a common comparative indicator, enabling us to relate changes in infrastructure, fishing capacity and fishing strategies to fluctuations in the fishery’s economy.

### 2. Historical data source

2.1 Fishery data: Landings and fleet composition

To reconstruct long-term patterns in sardine exploitation, we first compiled one dataset covering the historical period 1865–1965. This dataset systematized historical records in order to look for fluctuations in sardine abundance in the Bay of Biscay. Titled “Brittany_1865–1965_landings”, the data set is a collection of records on sardine landings derived from three different archives: Bibliothèque Nationale de France (1869, 1872–1875, 1880–1882, 1885–1906), Archives Nationales (1875, 1881–1882, 1898, 1900–1902, 1904–1905, 1910, 1913–1914), and Archimer (1866–1867, 1874, 1876–1880, 1883–1885, 1887, 1903, 1905, 1907–1909, 1911–1912, 1916–1965), which is the online archive of Ifremer (the French national research institute dedicated to the ocean and marine resources). The data set is composed of several time fragments, during which the recording methods and data organization remained consistent, facilitating intra-period comparisons despite an overall heterogeneity across the century (intra-periods 1865–1866; 1867–1874; 1875–1890; 1891–1893; 1894; 1895–1902; 1903–1912; 1913–1923; 1924–1937; 1938; 1945–1956; 1957–1958; 1959–1965).

These time fragments reflect the changing nature of data-collection practices over time. Throughout this century-long time span, the data show significant heterogeneity. Units of measurement vary from period to period, ranging from financial value (in francs) to the number of sardines caught, and later to weight—initially in kilograms and later in metric tons. For consistency, all quantities in weights have been standardized and expressed in kilograms. In some years, the official totals reported in the archival documents include additional fishing methods such as *pêche à pied* (literally “fishing on foot”, a practice dependent on the tides and the use of various tools) or distant-water expeditions (e.g. to Iceland or Newfoundland). These totals have been preserved in the dataset for each year and harbour to contextualize the relative importance of sardine fishing within overall landings. However, when isolating sardine catches specifically, only quantities caught through coastal operations were retained; for instance, sardines caught via *pêche à pied* were excluded from that column when identified. The data is organized differently across periods—sometimes by maritime district, sometimes by harbour—making direct comparisons challenging. From the late nineteenth century onward, additional details such as number of fishers, vessels, tonnage, fishing gear, and the seasonality of sardine fishing were progressively introduced. For the purposes of this study, only data relating to sardine fishing along the “Région de l’océan” (excluding the English Channel and North Sea) were retained.

2.2 Filtering the dataset

Since the dataset differed across periods, we standardized the temporal and spatial frames to ensure continuity and enable statistical analysis. The first filtered dataset, covering the years 1895 to 1912, includes information on the quantity of fish caught (in kilograms), the total value (in francs), the number of boats, and their tonnage capacity across fifteen districts: Arcachon, Audierne, Auray, Bayonne, Belle-Île, Camaret-sur-Mer, Concarneau, Douarnenez, Le Croisic, Lorient, Quimper, Saint-Gilles, Île d’Oléron, Noirmoutier, and les Sables-d’Olonne. The second filtered dataset spans a longer period but is limited to data on sardine landings: specifically, the kilograms caught and their value in francs. It includes the districts of Arcachon, Audierne, Concarneau, Douarnenez, Le Croisic, Lorient, and Saint-Gilles-sur-Vie.

### 3. Digitizing built environment data

3.1 Comparative mapping (1879)

We found on the online catalogue of the Archives Départementales du Finistère (ADF) scanned maps of fishing harbours in France. These maps were extracted from a book, *Atlas des Ports de France* (1879) written by the technical body of *Ponts et Chaussées*.

The urban plans are represented using a consistent scale and cartographic convention, allowing comparison of shoreline organization and maritime infrastructures. The maps were an entry point in examining how the terrestrial dimension of sardine exploitation evolved in relation to fishing intensity. We digitized and assessed these maps, with a focus on three sardine harbours in South Brittany: Douarnenez, Audierne, and Concarneau.

**Figure 1.**
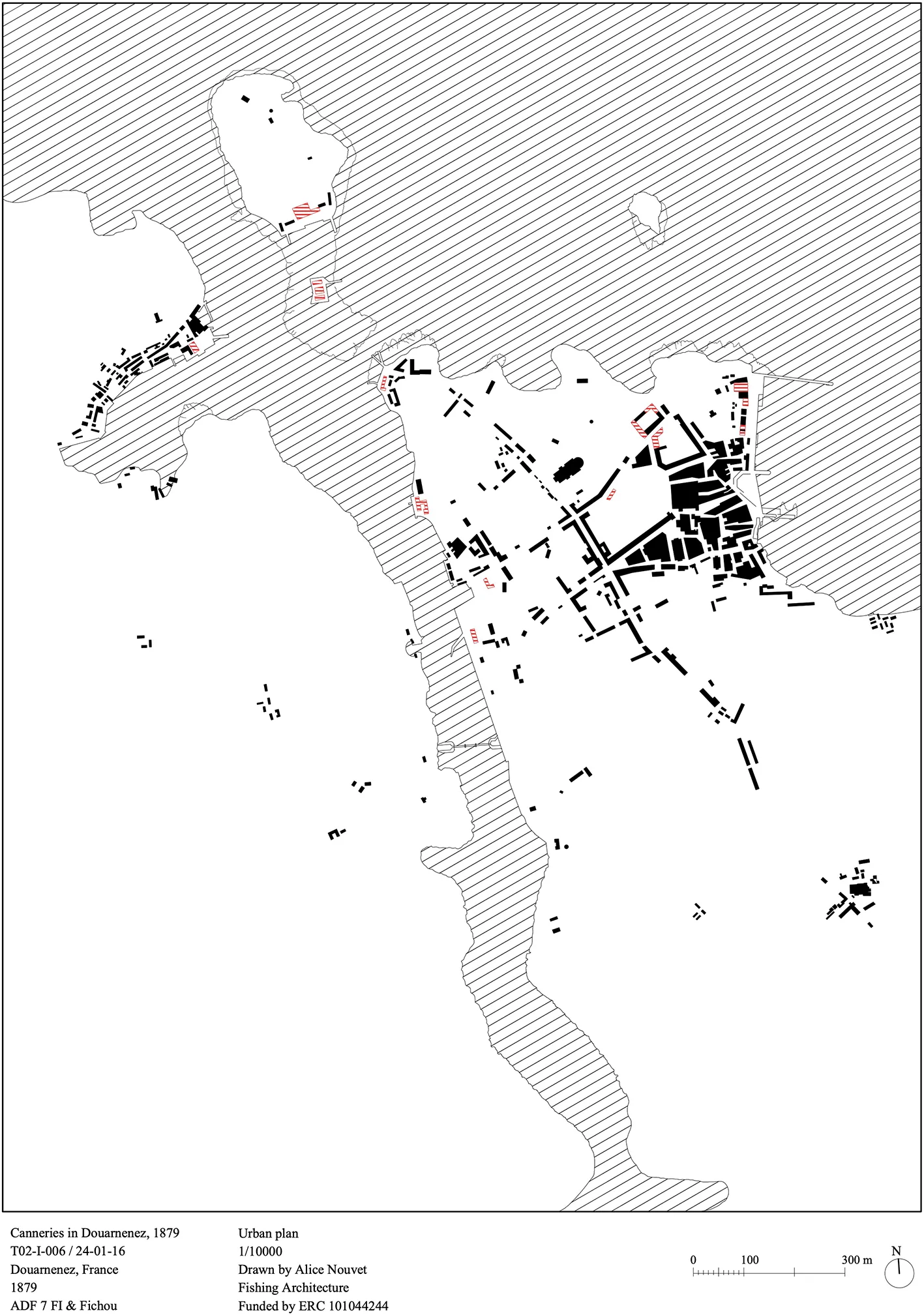
Urban plan, scale 1/10000, canneries of Douarnenez, 1879. Source: *Atlas des Ports de France*, Archives Départementales du Finistère, 7 FI.

The 1879 plans of Douarnenez, Audierne, and Concarneau were digitized as vectorial drawings on AutoCAD (such as Figure 1, representing only Douarnenez). These maps allowed us to delineate the main harbour docks and structures, such as slipways and breakwaters. We enriched these maps by locating canning factories as we cross-referenced maps produced by Jean-Christophe Fichou in his habilitation thesis (2006). This revealed how canneries positioned themselves within the harbour morphologies.

3.2 Temporal comparative mapping: Douarnenez, 1879 and 1909

**Figure 2.**
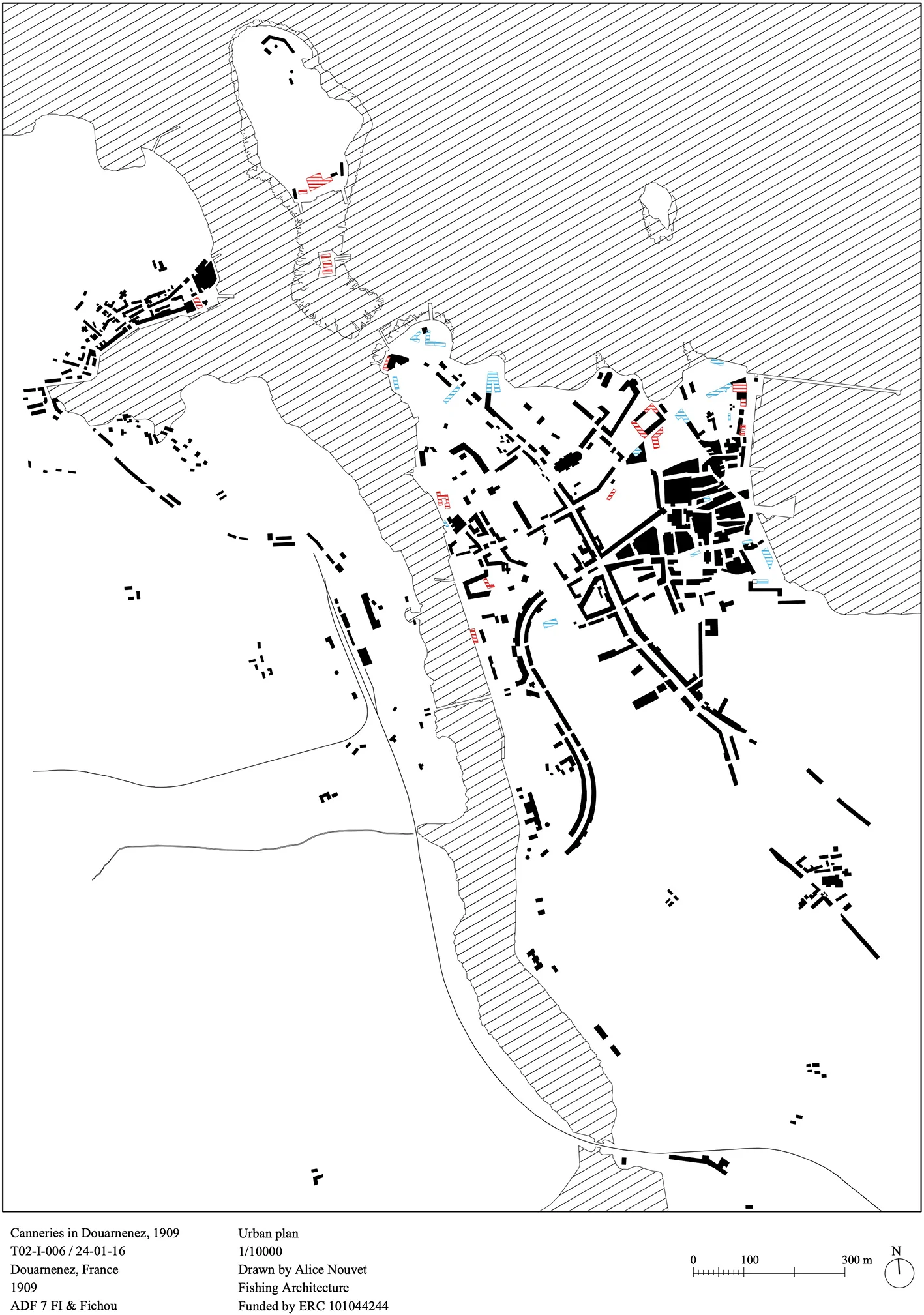
Urban plan, scale 1/1000, canneries of Douarnenez, 1909. Source: *Ports Maritimes de la France*, Service Historique de la Défense.

To understand change over time, we focused on Douarnenez, for which additional detailed materials were available. A 1909 urban plan found at the Service Historique de la Défense (SHD) in a book written by two *Ponts et Chaussées* engineers ( Corbeaux & Foucault, 1910), documents subsequent extensions and reconfigurations of the harbour areas, including the development of the Port de Rosmeur (fishing harbour) and Port-Rhu (commercial harbour). The construction of new docks and slipways, and the establishment of a bridge between Douarnenez and Tréboul to carry the railway ( Figure 2). This 1909 plan was digitized and processed following the same annotation procedure as for the 1879 series.

**Figure 3.**
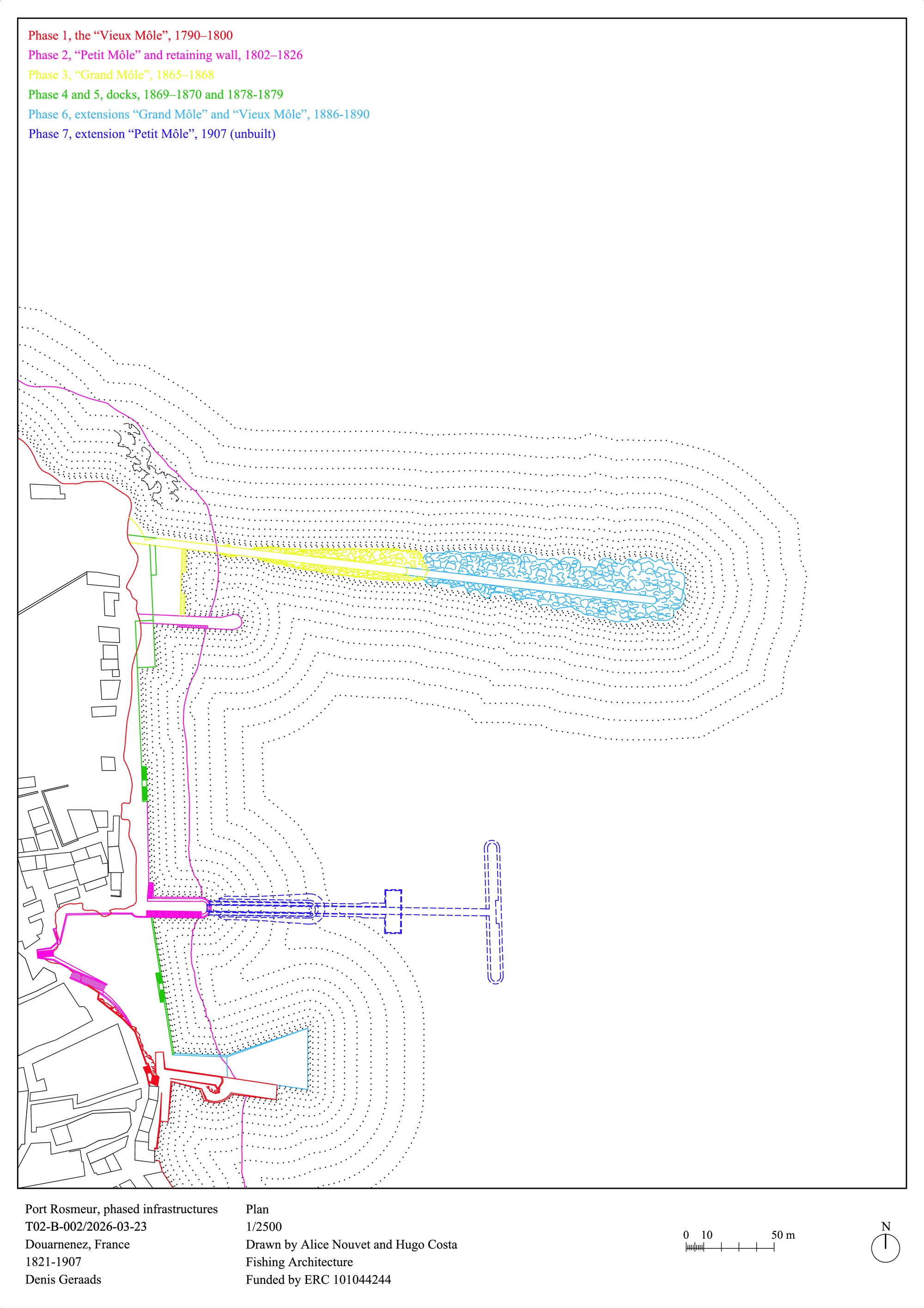
Urban plan, scale 1/2000, fishing harbor of rosmeur, Douarnenez. Phase 1, the “Vieux Môle”, 1790–1800; Phase 2, “Petit Môle” and retaining wall, 1802–1826; Phase 3, “Grand Môle”, 1865–1868; Phase 4, docks “Port de Rosmeur”, 1869–1870; Phase 5, docks “Grand Port”, 1878-1879; Phase 6, extensions “Grand Môle” and “Vieux Môle”, 1886-1890; Phase 7, extension “Petit Môle”, 1907. Source: Denis Geraads.

**Figure 4.**
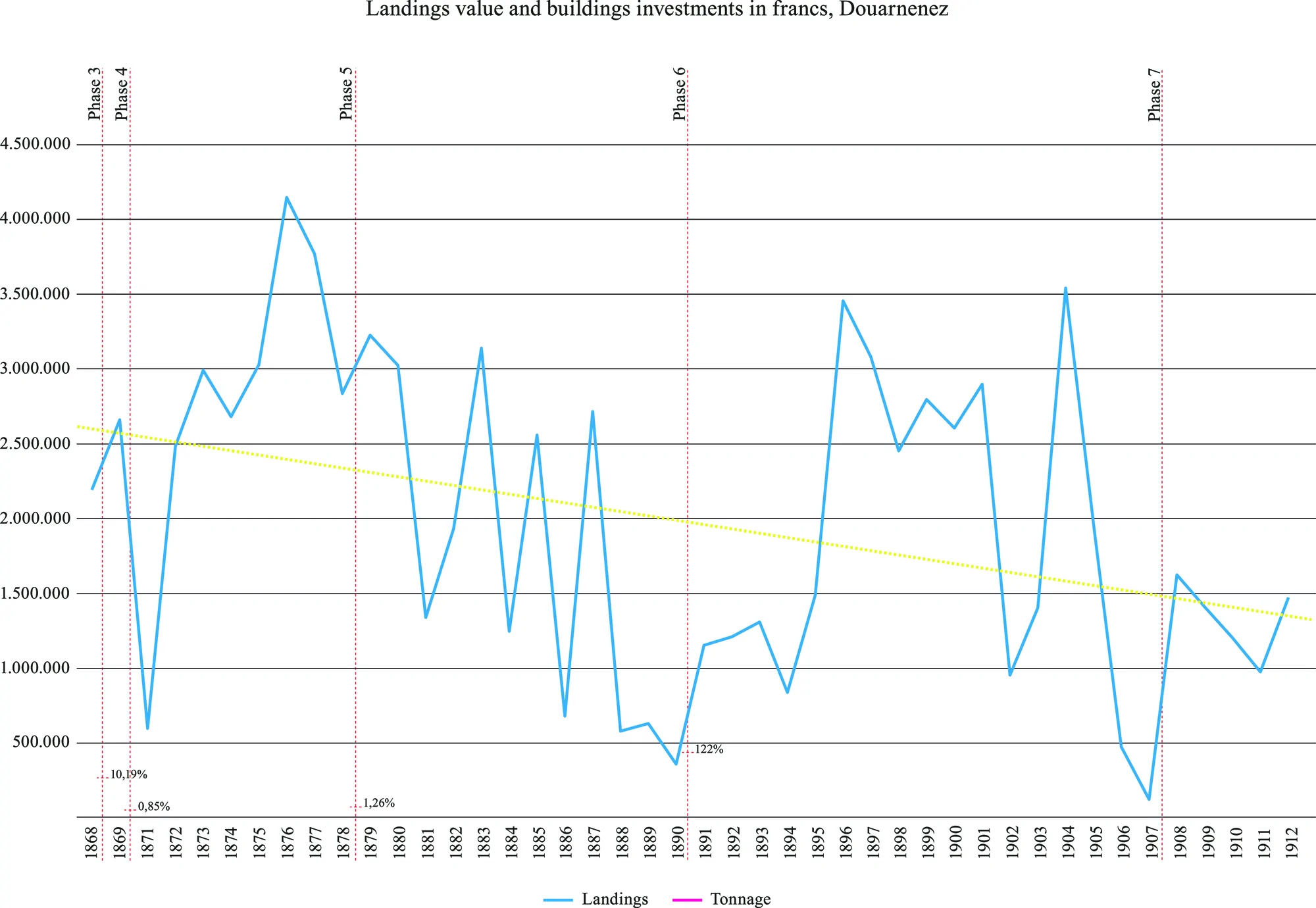
Graph with the values in francs of landings vs. phased investment harbour work. Source: Archimer and Service Historique de la Défense.

The 1910 *Ponts et Chaussées* book also provides quantitative data on harbour use and investments. This data inform us of the number of fishing boats entering and leaving Douarnenez between 1880 and 1906, as well as tables of construction works completed (piers, docks, slipways, etc.) and their related cost. From this material, we reconstructed a chronological sequence of the waterfront development in seven phases, between 1800 and 1907 ( Figures 3), indicating the economic value invested in each phase. It should be noted that fluctuations in the economic value of sardine landings do not directly reflect changes in catch volumes, as prices varied substantially depending on abundance, with scarcity driving prices upward and periods of abundance leading to price declines. An accompanying graph represents the value generated by sardine landings and how it was distributed across the phases ( Figure 4).

### 4. Statistical analysis

By using the Granger causality test ( Granger, 1969), a statistical method to determine if one time series can predict another time series, we analysed the time series of “landings” against “number of boats”, “tonnage” and “value”. The test assesses whether past values of one variable provide significant information about future values of another variable, indicating a predictive relationship rather than a direct causal one. The null hypothesis is that “landings” causes the “number of boats”, “tonnage”, or “values”. As the test is asymmetrical, the comparisons were performed in both directions. We used the datasets from the two continuous periods (1895–1912 and 1895–1938) for “values” and a continuous dataset from 1895–1912 for “number of boats” and “tonnage”.

We also analysed the influence of the environment and time over the “landings”, with a Generalized Linear Model (GLM) using the dataset of 1895–1912. We did not use mixed models as there are no repeated measures for each year. Hence, the variable “year” cannot be used as a random effect because mixed models require grouping and repeated measures: random effects are designed to model variability between groups when there are multiple observations within each group ( Wikle et al., 2019). Therefore, we compared landings against the environmental variables, such as sea surface salinity, sea surface temperature, surface upward latent heat flux, sea-level pressure, surface upwelling shortwave radiation, northward near-surface wind (respectively: sos, tos, hfls, psl, rsus, vas) and year as another fixed variable. We used a Gaussian distribution, as “landings” is a continuous variable. All variables were scaled, centred, and introduced in the model, as no variable pairs presented a correlation higher than 0.7 or a VIF (variable inflation factor) higher than 3 ( Field et al., 2012). We performed all analyses in R 4.5.1.

### 5. Continental shelf cartographies of the census and landings data

**Figure 5.**
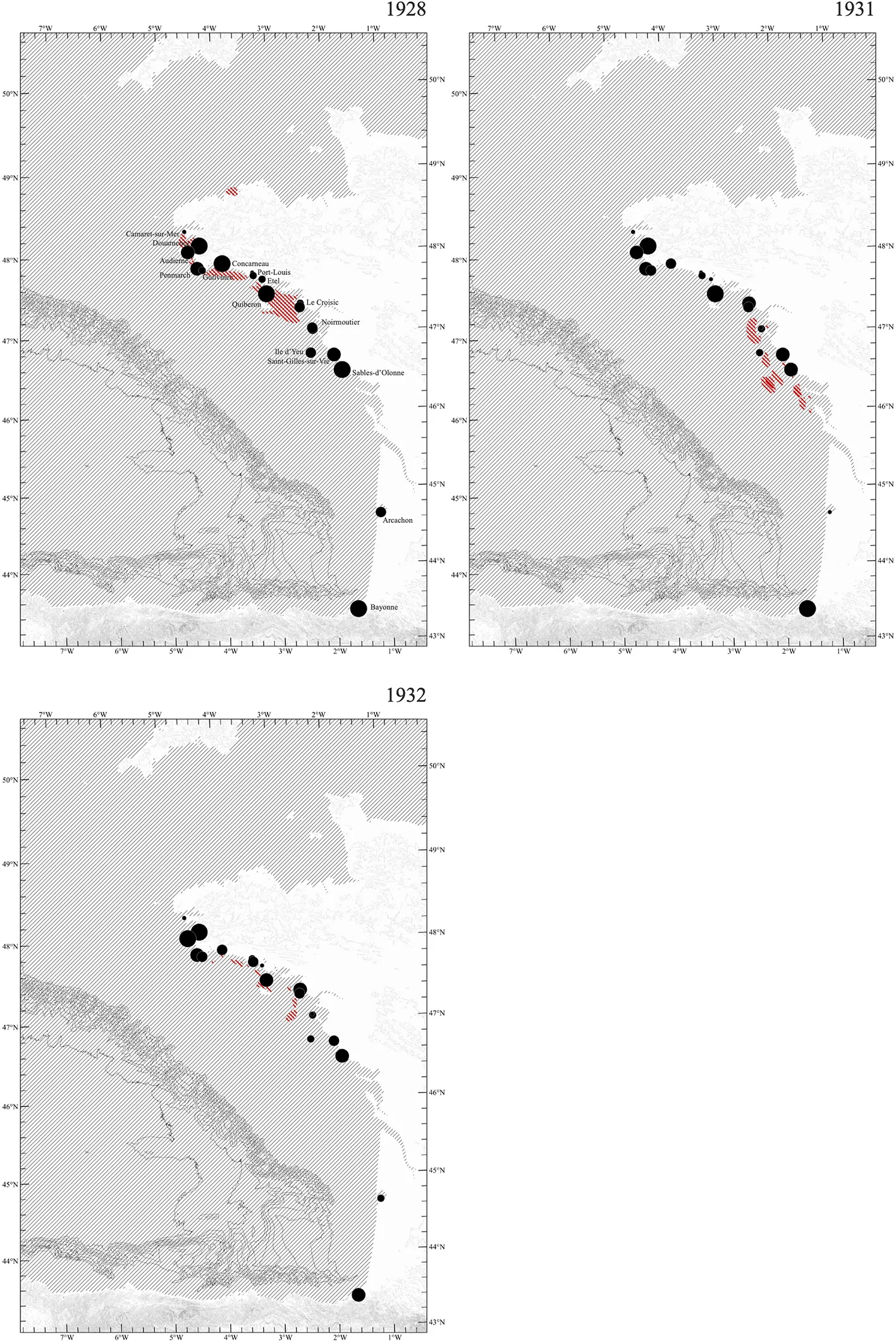
Series of cartography. Comparing fishing grounds and landings years 1928, 1931, and 1932. Source: Archimer.

A series of maps was created to represent the 1928, 1931, and 1932 fishing grounds, found respectively in the scientific reports of Jean Le Gall, Gérard Belloc, and Pierre Desbrosses ( Figure 5). The study of Jean Le Gall (1928) portrays the best-known fishing grounds along Brittany’s coast. He notes that sardine shoals appear later in the year as one moves northwards. According to Le Gall, this has led to a probably premature conclusion that the sardine is a migratory fish, moving from south to north. The study by Belloc (1932) followed the sardine fishing campaign of 1931 between the rivers Loire and Gironde. It also records the main fishing grounds. Belloc writes that each fishing ground is exploited for four to six months and observes the same pattern as Le Gall, with fish moving progressively from south to north. Desbrosses (1933) recorded the fishing grounds off the coast of Brittany in 1932. In each map, we juxtaposed the landing values per year to assess relations between the places and the numbers.

**Figure 6.**
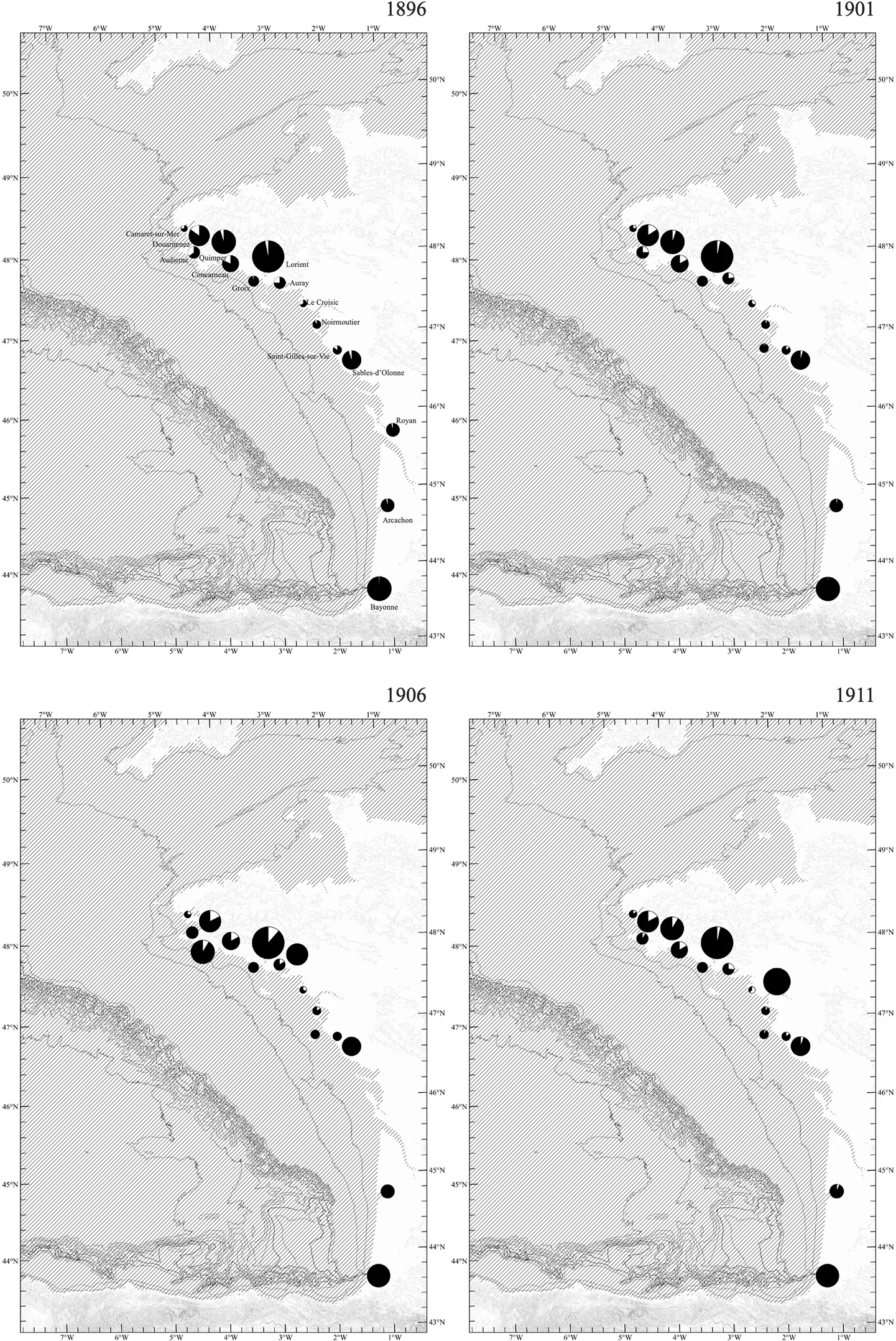
Series of cartography. Comparing fisher density within population density in fishing harbours, 1896, 1901, 1906, and 1911. Source: INSEE and Archimer.

**Figure 7.**
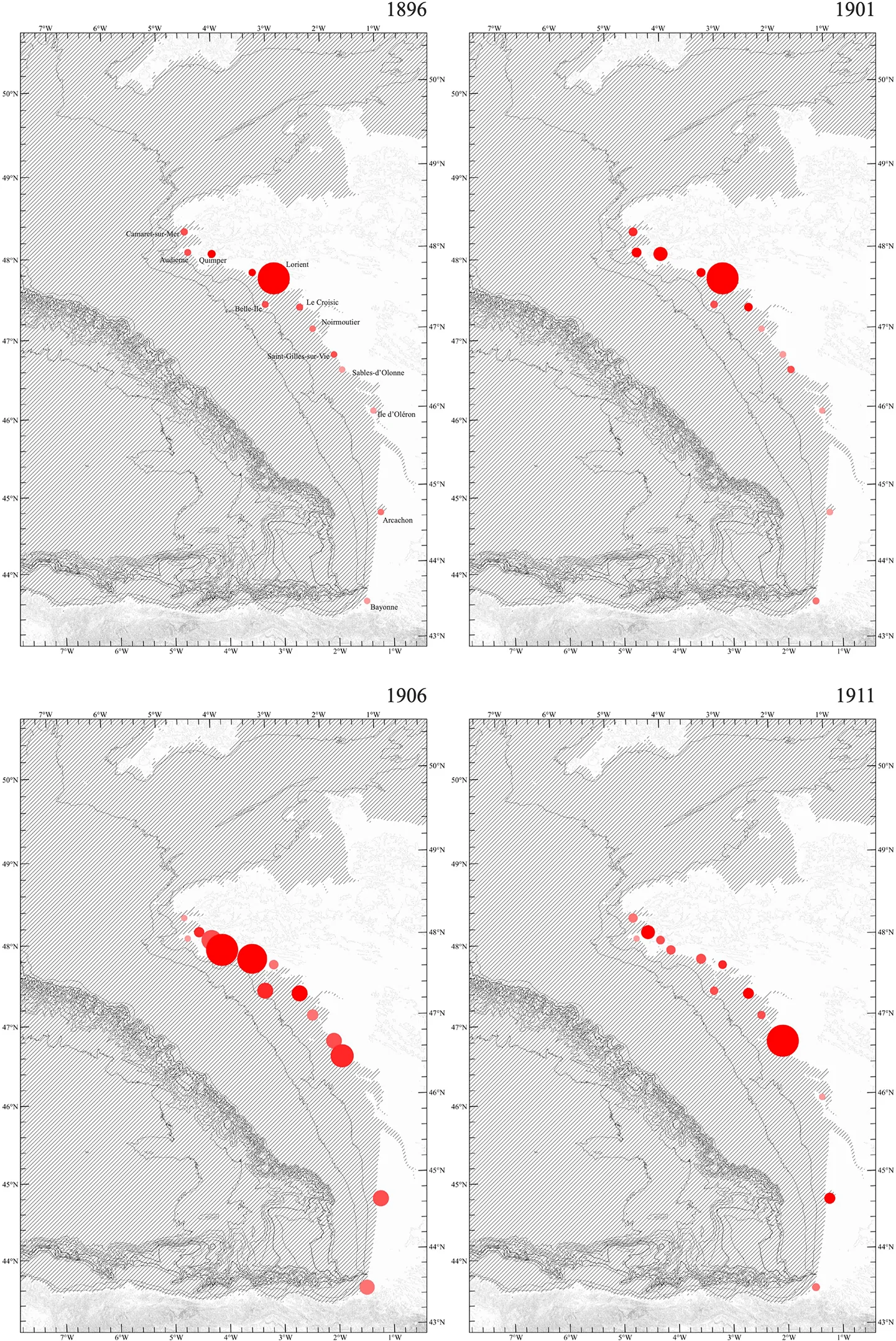
Series of cartography. Landings in kilograms and values, years 1896, 1901, 1906, and 1911. Source: Archimer.

Enlarging the frame of study, in order to address the development of coastal settlements, we assembled a dataset combining the number of fishers and the total population for each harbour on the total extent of the French Atlantic coast. This dataset drew on two sources: the population census from INSEE and the landings statistics, which include counts of fishers. Only harbours reporting the number of fishers were retained in the census subset. For each harbour, we calculated the proportion of fishers within the total population for the years 1896, 1901, 1906, and 1911 ( Figure 6). In parallel, we visualized the landings dataset (1895–1912) for the same four reference years (1896, 1901, 1906, and 1911), ensuring consistency with the census-based temporal slices. We restricted the representation to harbours for which comparable observations of both landings and fisher counts were available. Sardine revenues are represented through symbol colour, and landed quantities through symbol size ( Figure 7). Brest was excluded from the figure because it is only represented in 1896 and cannot be consistently integrated into the subsequent temporal comparisons.

## Results

### 1. Canneries’ urban occupation

Digitizing the built environment data of Audierne, Douarnenez, and Concarneau from 1879 allowed us to calculate the proportion of canneries within the total built-up area of each harbour. It indicated for Douarnenez a result of 6.19 per cent, for Concarneau 7.87 per cent and for Audierne 9.36 per cent. Yet, these percentages cannot be directly compared because the harbours differ in total built-up area, the absolute number of canneries, and their spatial distribution. The assessment was confined to the area covered by the *Ponts et Chaussées* drawings. For example, Audierne has the smallest built-up area (40,889 m² of urban footprint, including canneries, housing and other buildings) and the fewest canneries (five), followed by Douarnenez (90,925 m²; seventeen canneries), and Concarneau (110,269 m²; twenty canneries). Thus, while Concarneau has both the largest built-up area and the most canneries, Audierne has a higher proportion of its built environment devoted to canneries. Since there are no landings data, nor information on the workforce or vessels operating in these locations prior to 1882, our assessment is limited to the relationship between the urban footprint and the number and area of canneries.

The complementary materials we had for Douarnenez for the year 1909, allowed us to track its transformation between 1879 and 1909. A few aspects are very visible first hand: the arrival of the railway, the expanding built area (from 90,925 m ^2^ to 161,709 m ^2^) and the growing number of canneries (from seventeen to thirty-nine). Yet, the total annual value of sardine landings (expressed in current francs in the source statistics) declined by about half, from 3,208,579 francs in 1879 to 1,406,000 francs in 1909. This indicator reflects the aggregate monetary value of landings and therefore combines both changes in catch volumes and price fluctuations. This decline aligns with the literature on the sector’s economic crisis ( Dubois, 2004) and underscores the role of public investment in coastal infrastructures to mitigate it.

### 2. Shoreline infrastructures: value per square metre

The drawing representing the seven stages ( Figure 3) of coastal infrastructure in Douarnenez allowed to measure the surface of moles, docks, and slipways that were built to accommodate more fishing vessels. Only constructions expanding from the shoreline were measured. Due to inconsistencies in the representations of foundations and underwater works, our measurements were restricted to the visible surface elements. Hence, the figures represent minimum estimates of the real expansions. Having information on the value in francs invested for the building stages 3, 4, 5, and 6, we calculated the value per square metre of these infrastructures. The result was that for the establishment of the Grand môle between 1865 and 1868, with a surface of 1,785 m ^2^, the value per square metre was equal to 124.77 francs. The dock built to link the Grand môle to the Petit môle between 1868 and 1870, with a 1,702 m ^2^ surface, had a square metre value equal to 13.22 francs. The dock built to link the Petit môle to the Vieux môle between 1878 and 1879, with a surface of 3,409 m ^2^, had a square metre value of 10.41 francs. Finally, the two extensions of Grand and Vieux môle between 1886 and 1890 cost 221.39 francs/m ^2^. The variation is too large to be taken at face value. Moreover, moles required the extensive use of stone, whereas docks only needed a retaining stone wall and gravel and sand as fillers for the ground, thus becoming significantly less expensive.

### 3. Statistical analysis

The Granger causality test showed significant results only in the comparisons of “landings”, “tonnage”, and “values” ( Table 1). The comparison of “landings” and “number of boats” was marginally significant. The rest of the comparisons were not significant. Therefore, “values” depended on “landings”, and “landings” depended on “tonnage”. The number of boats has a minimum influence on landings as it mixes boats of different sizes: having more boats does not guarantee a larger catch. The GLMs comparing “landings” against the environmental variables and “year” for both periods reflected a lack of significance in almost all variables and intercepts ( Table 2): only the variable “year” and the intercept for the period 1895–1912 had a significant p-value (0.025).

**Table 1.** Granger causality test results. As the test is asymmetrical, the comparisons were performed in both directions: variable 1 vs. variable 2 and vice versa. DF is degrees of freedom; F is the statistical test metric. Significant p values are indicated with an asterisk (*).

Period	Variable 1	Variable 2	DF	F	p-value
1895–1912	Landings	N boats	−1	4.8126	0.046
1895–1912	N boats	Landings	−1	0.2275	0.641
1895–1912	Landings	Tonnage	−1	5.5799	0.033*
1895–1912	Tonnage	Landings	−1	0.4537	0.512
1895–1912	Landings	Values	−1	0.2178	0.648
1895–1912	Values	Landings	−1	0.1468	0.707
1895–1938	Landings	Values	−1	1.112	0.298
1895–1938	Values	Landings	−1	5.8256	0.020*

**Table 2.** Results of the generalised linear models (GLM) between landings and environmental variables for the periods 1895-1912 and 1895-1938. It is indicated the estimate, the standard error, the value of the statistic T, and the p-value. sos: Sea Surface Salinity; tos: Sea Surface Temperature; hfls: Surface Upward Latent Heat Flux; psl: Sea Level Pressure; rsus: Surface Upwelling Shortwave Radiation; vas: Northward Near-Surface Wind.

Period 1895–1912				
Variable	Estimate	Standard error	T value	p-value
Intercept	205.1879	78.0536	2.629	0.025*
sos	−0.0702	0.2423	−0.289	0.776
tos	0.2762	0.2417	1.143	0.280
hfls	0.1363	0.2980	0.457	0.657
psl	0.0183	0.3528	0.052	0.960
rsus	−0.2260	0.2827	−0.800	0.443
vas	−0.3933	0.2762	−1.424	0.185
year	−0.1078	0.0410	−2.629	0.025*

Period 1895–1938				
Variable	Estimate	Standard error	T value	p-value
Intercept	37.4562	30.7625	1.218	0.231
sos	0.0239	0.1647	0.145	0.886
tos	0.1587	0.1953	0.813	0.422
hfls	−0.0221	0.1903	−0.116	0.908
psl	−0.0071	0.2617	−0.027	0.979
rsus	0.0603	0.1997	0.302	0.765
vas	−0.2751	0.1818	−1.513	0.139
year	−0.0195	0.0161	−1.218	0.231

### 4. More fishing pressure in the *plateau armoricain* than the *plateau aquitain*?

There is a similar spatial distribution between the 1928, 1931, and 1932 maps of fishing grounds ( Figure 5). In all three years, fishing pressure was concentrated on the *plateau armoricain* and limited to areas shallower than the 50-metre bathymetric line. This may partly result from a bias in the available sources, which are available for this region and may unbalance the figures between north and south. However, the same imbalance also appears in the national landings dataset and fishermen census, which shows a concentration of fishing activity along the coast facing the *plateau armoricain.*

The census and sardine landings datasets assembled during this research share data for eleven harbours in four years (1896, 1901, 1906, and 1911). Within the *plateau aquitain*, only two coastal towns, Arcachon and Bayonne, appear in the dataset. In these, the proportion of fishers within the total population is extremely small and barely visible in the figures, though present in the census records (Bayonne: 1886, 0.25%; 1901, 0.55%; 1906, no info; 1911, 0.38%; Arcachon: 1886, 0.29%; 1901, 0.60%; 1906, no info; 1911, 1.31%). Landings data reflect the same trend, showing very low sardine volumes and economic value. By contrast, the coastal towns bordering the *plateau armoricain* present higher proportions of fishers (often approaching one quarter of the total population) and their landing records show a corresponding concentration of sardine activity.

### 5. Continuous average decline and public investment

**Figure 8.**
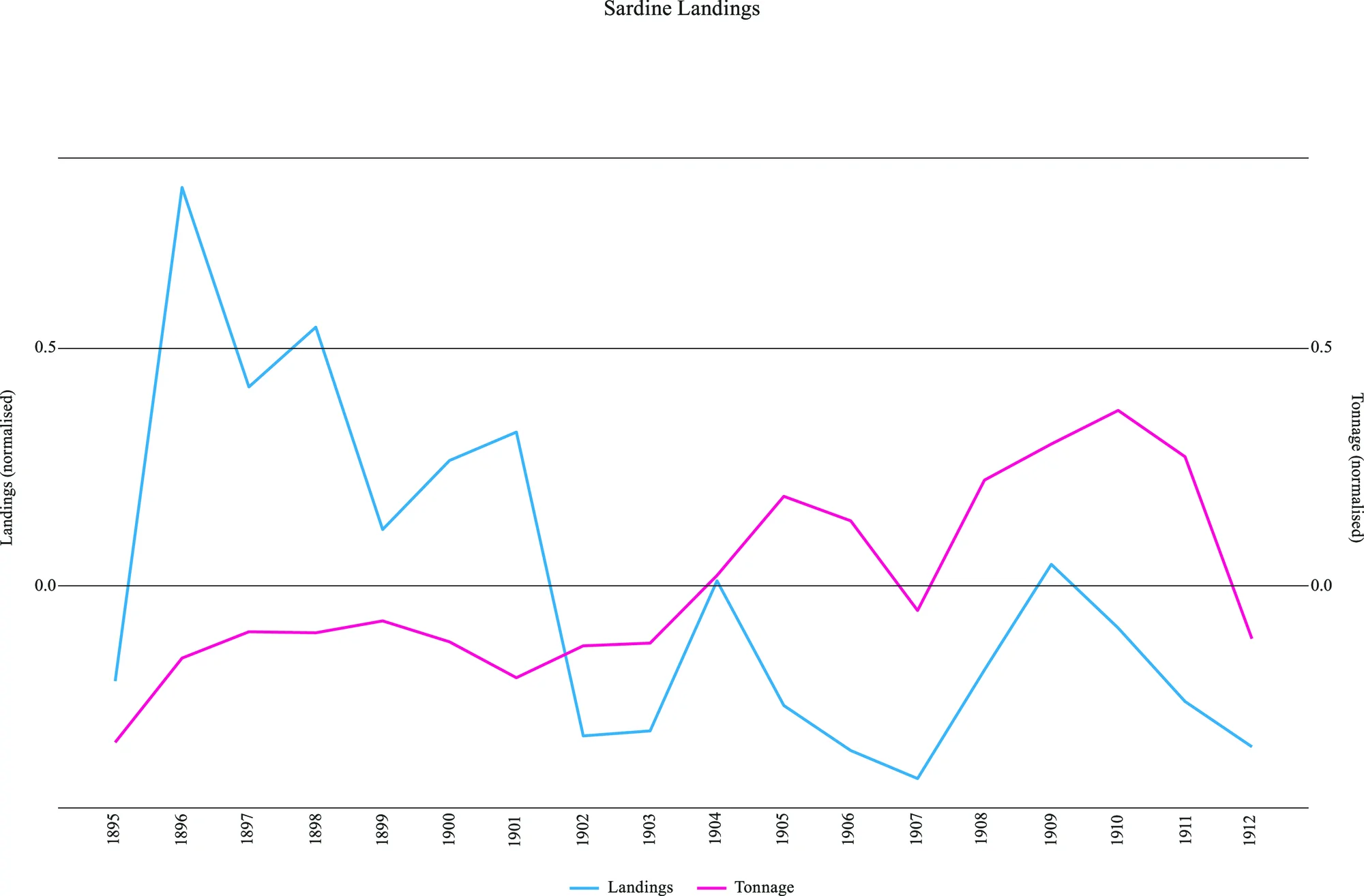
Graph comparing landing values and vessels tonnage. Source: Archimer.

The clearest result of the data analysis was the continuous decline of the landings throughout the study period (see trend line in Figure 4). Major public investments were deployed both for vessels ( Figure 8) and coastal works after or during years of significant fisheries collapse. In general, they recovered the sardine economy, but profits remained weaker than in previous years. It is also blatant that the public sums invested represented a tiny percentage of the global sardine business value. Since we did not obtain the years nor the values of private investment in construction and transformation of canneries and their equipment, there was no comparison possible between public and private investment within the sardine economy of the period.

## Discussion

The results show three broad patterns: a long-term decline in sardine landings that does not seem to be the influenced by environmental variability; the continuing expansion of harbour infrastructures and vessels tonnage even during periods of fluctuating catches; and a spatial concentration of fishing effort on the *plateau armoricain.*

### 1. Latency and misalignment in harbour work

Built forms such as moles, breakwaters, slipways and docks organized the circulation of vessels, fish, and labour, thereby structuring how value could flow between marine ecosystems and terrestrial landscapes. However, the relationship between architectural investment (both in vessels and coastal works) and sardine landings appears oblique: construction works often occurred shortly after periods of decline in landing values. While we cannot state that these were intentional responses, this temporal coincidence suggests that, in the case of harbour facilities, public investment took place during periods of social unrest and instability within the sardine economy. These investments may therefore be understood as part of a broader state effort to stabilize and maintain fishing activities, rather than profit-driven actions. Yet, the Ponts et Chaussées book ( Corbeaux & Foucault, 1910) shows us that the harbour work followed an open and pre-established plan drawn up by administrative bodies as part of long-term programmes, which reinforces the idea that investments did not necessarily match the realities of the fisheries.

The plans made by the *Ponts et Chaussées* did not introduce fixed forms, only proposals as dotted lines, with scope for adaptation. These proposals were sometimes abandoned or postponed and picked up years later by the new engineers in charge. For example, the sequential transformations of Port de Rosmeur were dictated by constant programmes of repair. As Zitouni & Tellier (2013) argue, the dotted lines in the drawings are a deliberate strategy of latency: by inscribing possible futures in advance, the technical body delimits and conditions the range of transformations that will later “appear feasible”, thereby producing a future that is already partly foreclosed. This insight is particularly relevant as these pre-shaped harbour configurations ended up creating the conditions in which fishing pressure on sardine populations intensified. And ultimately, the “open plan” left a long-term mark within the territory: more than a century later, what can be seen today in Douarnenez’s shore is the result of the successive milestones chosen and designed by the *Ponts et Chaussées.*

### 2. Anthropic drivers of sardine landings

The statistical analyses show that the variable “landings” were influenced by the variable “tonnage” for the period of 1895–1912. And the variable “values” were influenced by the variable “landings”. Landings increased because the boat capacity increased as well, and values increased with landings over time. In our analysis, the “landings” were not influenced by the environmental variable; thus, the changes in the landings over time were influenced by anthropic pressure rather than climatic factors. In fact, after strong decreases in landings, the increase in boat capacity (“tonnage”) made landings bounce back, although the quantity did not get back to the initial values of the period ( Figure 4). The lack of relationship between landings and the number of boats confirms that the influencing factor was the increase in boat capacity (“tonnage”) and not the increase in the number of boats, which might include small and large fishing boats. There was an important but temporary increase in the number of boats after 1905, but that did not translate into a substantial increase in landings. Profits (“value”) substantially increased after 1920, despite the small increase in landings. And the fish price increased because fish became a rare commodity. This last aspect is supported by existing literature ( Dubois, 2004).

As the landings data does not directly reflect the actual abundance of European sardine populations in the Bay of Biscay but rather serves as a proxy in our analysis, we cannot rule out the possibility that the fish had simply moved further offshore, beyond the reach of the fishing vessels. In this case, the fishing vessels may have continued to intensively exploit nearshore areas that were already being deserted by migrating sardines.

Moreover, while the influence of tonnage on landings does not present itself as a causality, it does point to a relationship in which coastal architectures and vessels may have reinforced each other over time. Larger vessels required deeper mooring and extended moles, and, in return, harbour expansions facilitated more and larger vessels operations ( Fichou, 2003).

### 3. Architecture as a material conduit

The figures representing the landings across time show that they did not follow a consistent spatial distribution, which could be explained by the movement of sardines. Yet, existing literature suggests an interesting hypothesis: landings were often concentrated in harbours with favourable infrastructure, more accessible docks, and multiple canneries, regardless of their proximity to rich fishing grounds ( Fichou, 2006). This spatial mismatch highlights the influence of industrial geographies for fishers at sea. Fishers landed where processing capacity, buyers, and harbour facilities could handle their catch with certainty. Architecture, in this sense, greatly influenced the economic system of sardines and the locations where economic opportunities materialized. It did not determine an immediate increase in catches, but it shaped when and where sardines entered the economic circuit, with architecture becoming a material conduit for sardine value.

### 4. Following sardines’ skin

The figures for landings and fishing grounds reinforce the idea of architecture as a material conduit as they reveal an odd imbalance between south and north. Early observers interpreted this spatial pattern as a south–north sardine migratory movement ( Un pêcheur, 1864), linking the progression of the fishing season to the movement of both fisherfolk and sardines. The sardine fishing season opened between February and March on the southern coasts and advanced northward over the course of the year, ending in October/November in the bay of Douarnenez. Fishing activities shifted accordingly, with boats and crews moving from harbour to harbour as catches declined in the south and increased further north. This displacement of fishing effort was understood as the result of sardines themselves migrating northward, growing and changing as they moved. This interpretation, based on the directional migratory pattern, is no longer supported today. Sardine movements are now understood as responses to environmental conditions that shape spawning and feeding areas ( Bellier et al., 2007; Planque et al., 2007). Sardines are opportunistic feeders and batch spawners, reproducing through successive spawning events rather than in a single episode ( Dessier, 2015). Spawning and feeding zones may therefore overlap at certain times while remaining separate at others. This could help explain earlier observations of mixed size classes within the same fishing grounds, where larger and smaller sardines were sometimes seen together, without implying a linear growth trajectory from south to north.

However, in De la pêche de la sardine et des industries qui s’y rattachent ( 1864), the description of sardines’ skin provides an explanation for the northward concentration of canning factories. The text describes southern sardines as small, tender, fragile fish, with soft skin and flesh that made them difficult to conserve—particularly when it came to preserving the oil. Further north, sardines are described as larger and firmer, reaching what was considered an optimal state for the canning industry, especially between Concarneau and Douarnenez. Canneries required sardines that met both size and texture thresholds—neither too small and fragile, nor too large and oily—thus, fishing pressure tended to concentrate in areas where these conditions were most often encountered.

If these observations accurately reflect the requirements of the nineteenth-century canning industry, they also emphasize that the geography of fishing harbours cannot be understood solely through the physical characteristics of the coastline. The northern shoreline is generally more indented and rockier than the beach-dominated southern coast, suggesting that fishing infrastructures may have been more driven by the sardine skin qualities sought by canneries.

### 5. Limitations

Because preliminary archival work across municipal, regional, and national archives was constrained by time, additional sources are still to be found and may yet complement the datasets assembled here. More continuous records of coastal transformations, together with documentation of other building typologies (such as salt depots, auction houses, shipyards, etc.) and information on private investments in canning factories, would allow a systemic reconstruction of the economic and political choices that shaped the sardine industry. Such materials could extend the research on Douarnenez and allow the methods to be transposed to other fishing harbours. This would reduce the reliance on population censuses as proxies for fishing activity. Nevertheless, further archival discoveries are unlikely to resolve the principal limitation of this study: the absence of historical scientific observations capable of reconstructing sardine abundance independently from landing records.

## Conclusion

The materials collected in archives pushed this work to engage with two complementary scales: a detailed analysis of a single harbour and a broader comparison across multiple fishing towns distributed along the continental shelf of the Bay of Biscay. The evidence assembled here reveals several important patterns. First, the geography of sardine fishing harbours cannot be explained solely on the basis of the physical characteristics of the coastline. The concentration of canneries and harbour infrastructures along the rocky shores of Brittany is not self-evident when compared with the sandy coasts further south. Historical descriptions repeatedly distinguished sardines according to their size, texture, and skin quality, describing the sardines of Brittany as particularly suited to the requirements of the canning industry. If these observations accurately reflect industrial preferences, the geography of fishing infrastructures was shaped not simply by the natural features of the shore but also by the qualities of the fish themselves. In this sense, the geography of the sardine’s architecture followed the fish before it followed the shore.

Second, the comparison of fishing grounds, landing records, and harbour developments suggests a distinction between where sardines were caught and where their economic value was realized. Fishers continued to follow productive fishing grounds, but they did not necessarily land their catches in the nearest harbour. Instead, catches tended to be landed in harbours equipped with moles and canneries capable of purchasing and processing the fish with certainty. In other words, the geography of fishing grounds did not fully determine the geography of landings. Architecture therefore acted as more than a passive backdrop of fisheries. Equipped harbours attracted and channelled catches from a wider maritime territory.

Third, the study documents a persistent divergence between declining landings and continued material investment. Sardine landings followed a long-term downward trend throughout the period under study, yet vessel tonnage increased and harbour infrastructures continued to expand. The evidence suggests that these interventions formed part of broader efforts to sustain regional economies under pressure. In the case of harbour infrastructures, many projects had already been anticipated within the plans of the Ponts et Chaussées and were subsequently activated, resumed, or accelerated during periods of instability. The built environment therefore responded less to immediate ecological conditions than to longer-term development trajectories embedded within administrative and technical practices.

Taken together, these findings point toward the coexistence of multiple temporalities within the sardine fishery. Sardines responded to daily movements through the water column, seasonal cycles, and longer-term oceanographic variations. Fishing grounds shifted according to interannual and multidecadal environmental conditions. Vessels were repaired, enlarged, and replaced over successive decades, while harbour infrastructures and canneries were conceived as investments intended to endure for generations. The concentration of canneries in Brittany, the attraction exerted by equipped harbours, and the persistence of investment despite declining landings can all be understood as consequences of interactions between processes operating at different speeds.

By bringing together marine biology and architectural history, we can understand how ecological and material landscapes jointly produce each other and how their histories continue to structure coastal territories today.

## Ethics and consent

Ethical approval and consent were not required.

## Data Availability

Zenodo. Three Historical Data Sets on Sardine ( *Sardina pilchardus*) Fisheries and Climate in the Bay of Biscay (1846–1965): Landings, Vessels, and Environmental Variables. https://doi.org/10.5281/zenodo.15719564 ( Nouvet, A., et al. 2025). This project contains the following underlying data:
• Brittany_1865-1965_landings.xlsx (This is an excel file on European sardines ( *Sardina pilchardus*) landings on the Atlantic coast of France, from Brest to Saint-Jean-de-Luz, between 1865-1965) • Brittany_1865-1965_landings_and_climate.xlsx (This is an excel file on European sardines ( *Sardina pilchardus*) landings, from Brest to Saint-Jean-de-Luz and climate variables of the Bay of Biscay between 1865-1965) • Brittany_1846-1928_sardine_fishing_vessels.xlsx (This is an excel file on the launch and years of operations of sardines fishing vessels in South Brittany between 1847-1928) • netcd2tif_v4.py (This is the python script to read the CMIP6 netCD files) •
year_sos_tos_table.R (This is R script to extract sea surface temperature and salinity variables) •
year_winds_table.R (This is R script to extract wind related variables) Data is available under the terms of the CC-BY-4.0
