# Supplementary material for: Histories of sardine fisheries. architecture and environment in the Bay of Biscay (1846–1965)
Source: Open Res Eur. 2026 Jul 14;6:101. Originally published 2026 Apr 10. [Version 2] doi: 10.12688/openreseurope.23094.2 (PMC13554732; doi:10.12688/openreseurope.23094.2)
Supplement: Peer review history

# Reviewer report: Reviewer response for version 2

**DOI:** 10.21956/openreseurope.26455.r77427

**Published:** 2026-07-23

**Authors:** Jack Bouchard

**Affiliations:** 1 Rutgers University, New Brunswick, NJ, USA

I think that the revisions are effective and address my main concerns. I yield to the authors regarding the scope and goals of the piece, which are better articulated in this version. I think it should be indexed as it is and thank the authors for producing such an interesting study.

If any, are all the source data and materials underlying the results available?

Yes

Is the work original in terms of material and argument?

Yes

Is the work clearly and cogently presented?

Partly

Is the argument persuasive and supported by evidence?

Yes

Does the research article contribute to the cultural, historical, social understanding of the field?

Yes

Does it sufficiently engage with relevant methodologies and secondary literature on the topic?

Yes

Reviewer Expertise:

Fisheries history, premodern (XV-XVII centuries); Marine environmental history

I confirm that I have read this submission and believe that I have an appropriate level of expertise to confirm that it is of an acceptable scientific standard.

# Reviewer report: Reviewer response for version 1

**DOI:** 10.21956/openreseurope.24982.r72270

**Published:** 2026-05-11

**Authors:** Christy Anderson

**Affiliations:** 1 University of Toronto, Toronto, Ontario, Canada

The most original claim in Nouvet et al. is that architecture is not a passive backdrop to fisheries history but a "material conduit" through which sardine value flowed and through which fishing pressure was channelled. The piers, moles, slipways, docks, and cannery footprints of Douarnenez, Audierne, and Concarneau organised where fish entered the economic circuit, and the seven-phase reconstruction of the Port de Rosmeur shows with quiet authority how state engineering practice accumulated, decade by decade, into the shoreline we still see today. This is a genuinely architectural argument made on architectural evidence, and it deserves to be defended as such. The reviewer is right that the paper's hedging language ("to a certain extent we can argue," "we discovered that this was not necessarily the case") undercuts the force of the finding. The finding itself is bold: the built environment shaped the sardine economy more decisively than the sardines did.

 What weakens the architectural argument is not its substance but its narrow material range. The paper treats moles, docks, slipways, and canneries, the heavy stone, brick, and timber fabric of the harbour edge, but stops there. Sardine fishing was made possible by a much wider architectural and material world that the paper leaves out: the seines and drift nets, the cordage and rigging, the tarred lines, the wooden hulls and iron fastenings, the salt and oil and tin and solder that moved through the canneries, and the women who worked them. Each of these had its own architectures on land. Ropewalks, sail lofts, cooperages, tin-plate works, salt depots, and fish-meal sheds were as terraqueous as the moles. To argue that architecture organised the sardine economy is to argue that this whole infrastructural fabric, not just its masonry edge, did the organising. Alison Bashford's "Terraqueous Histories" ( *The Historical Journal*, vol. 60, no. 2, 2017, pp. 253–272) is useful here only because she names the conceptual move the paper is already making, collapsing the land/sea couplet into a single material world. The architectural case stands on its own, and would stand more firmly if the building types under analysis were broader.

 The strongest single observation in the paper is that infrastructure investment continues, and even accelerates, while landings decline. This is an architectural finding with serious implications for how we read coastal landscapes. The Ponts et Chaussées drawings, with their dotted lines and phased proposals, show that the harbour was never the product of any one moment's economics. The mole built in the crisis of 1886 had been imagined twenty years earlier and would shape the harbour for a century after. Architecture on the shore therefore operates at a different temporality from the sea it serves. Sardines move in seasons; vessels in years; landings in decades; but moles and canneries in centuries. The paper has the evidence to make this temporal argument explicit and is one rewrite away from doing so. The reviewer's call for "clearer, more forceful argumentative language" applies most of all here.

 What the architectural framing also makes possible, and what the paper has not yet claimed, is a connection between the building on land and the vessel at sea. The Granger test result that landings depend on tonnage is not merely a statistical finding; it is an architectural one. Larger vessels needed deeper moorings, longer moles, wider slipways, and the harbour transformations of the 1880s and 1890s answered those needs while also enabling the next generation of larger vessels. The mole and the boat shaped each other. This reciprocal building, on water and on land, is what gives the paper its claim to a maritime architectural history rather than a coastal one. The vessels dataset that the authors compiled but barely use (1846 to 1928) is the bridge they have not yet crossed: the boat is also a building, and reading the boat alongside the mole would let the architectural argument finally span the shoreline.

 Bashford's "terraqueous" is one term for what this paper is reaching toward, and the reviewer is right to feel that something like Westerdahl's Maritime Cultural Landscape is missing. But the deeper point is that the architectural history of the sea is still being written, and Nouvet et al. have made a real contribution to it. Their evidence (the 1879 atlas, the 1909 plan, the seven-phase reconstruction, the cannery footprints, the vessel registers) is exactly the material from which such a history must be built. What the paper needs is the confidence to claim that ground openly: to treat the architecture of the harbour, the cannery, the ropewalk, and the vessel as a single object of study, and to argue that this architecture, not the fish and not the climate, organised the sardine century.

If any, are all the source data and materials underlying the results available?

Yes

Is the work original in terms of material and argument?

Yes

Is the work clearly and cogently presented?

Yes

Is the argument persuasive and supported by evidence?

Yes

Does the research article contribute to the cultural, historical, social understanding of the field?

Yes

Does it sufficiently engage with relevant methodologies and secondary literature on the topic?

Yes

Reviewer Expertise:

architectural history, early modern maritime history, European trade and economic history

I confirm that I have read this submission and believe that I have an appropriate level of expertise to confirm that it is of an acceptable scientific standard.

# Response

**Published:** 2026-06-25

**Authors:** Alice Nouvet

**Affiliations:** Universidade do Porto Faculdade de Arquitectura, Porto, Porto District, Portugal

Dear Reviewer, We are grateful for the careful reading of the manuscript and the insightful and constructive comments. The remarks have been helpful in clarifying and strengthening the framing of our contribution.

**Comment 1:** “The reviewer is right that the paper’s hedging language (‘to a certain extent we can argue’, ‘we discovered that this was not necessarily the case’) undercuts the force of the finding. The finding itself is bold: the built environment shaped the sardine economy more decisively than the sardines did. (…) What the paper needs is the confidence to claim that ground openly: to treat the architecture of the harbor, the cannery, the ropewalk, and the vessel as a single object of study, and to argue that this architecture, not the fish and not the climate, organized the sardine century.”

**Response: **We appreciate this observation and agree that our earlier wording may not have sufficiently clarified our position regarding the relationship between sardines and coastal architectures. Our aim was not to adopt a framework in which architecture alone determines the organization of the sardine economy independently of marine life. Instead, our approach explores the correlations between ecological and material landscapes. The introduction of the notion of “sardine skin” in the discussion section of the revised manuscript is intended to address this point. Historical sources consistently describe how variations in the size, texture, and skin quality of sardines influenced their suitability for canning and therefore affected the spatial distribution of landing and processing activities. Sardines participate in shaping coastal infrastructures as biological entities whose qualities actively condition industrial choices.

**Comment 2:** “What weakens the architectural argument is not its substance but its narrow material range. The paper treats moles, docks, slipways and canneries, the heavy stone, brick and timber fabric of the harbor edge but stops here. Sardine fishing was made possible by a much wider architectural and material world that the paper leaves out: the seines (…). To argue that architecture organized the sardine economy is to argue that this whole infrastructural fabric, not just the masonry edge, did the organizing.”

**Response:** Regarding the conceptual framing of architecture and its relation to wider maritime material artefacts, we fully agree with the observation that the infrastructures discussed in the paper exist within a broader terrestrial and maritime assemblage. In the revised version, we state more explicitly that these elements are part of a broader maritime material world, both in the objectives and in limitations sections. Nonetheless, they are not the primary objects of analysis in this paper. This is not meant to exclude their importance but is rather a methodological delimitation necessary to maintain a coherent and comparable longitudinal dataset focused on harbor-based infrastructures, which are the elements consistently traceable across space and time in our sources.

**Comment 3: **“Sardines move in seasons; vessels in years; landings in decades; but moles and canneries in century. The paper has the evidence to make this temporal argument explicit and is one rewrite away from doing so.”

**Response:** We are grateful for the remarks on temporality, which proved particularly helpful in clarifying a dimension that had remained implicit in earlier versions of the manuscript. As observed, the argument gains strength when the different temporalities at play are made explicit. The revised conclusion now explicitly distinguishes ecological, operational, and infrastructural temporalities. The way the review addressed this issue was especially helpful in guiding this revision, and we have used it to hone the argument that architectural operations often persist independently of ecological fluctuations.

**Comment 4:** “What the architectural framing also makes possible, and what the paper has not yet claimed, is a connection between the building on land and the vessel at sea. The Granger test result that landings depend on tonnage is not merely a statistical finding; it is an architectural one. (…) The vessels dataset that the authors compiled but barely use (1846 to 1928) is the bridge they have not yet crossed: the boat is also a building, and reading the boat alongside the mole would let the architectural argument finally span the shoreline”

**Response:** We agree that the vessel dataset provides an important link between terrestrial infrastructures and fishing activity at sea, and that the relationship between vessel capacity and harbour development deserved further attention. In response, we have substantially expanded our use of the vessel dataset (1846–1928). A new figure now compares vessel tonnage with the economic value of sardine landings (figure 5), complementing the existing analysis of harbor-work investments. This comparison reveals a pattern similar to that observed for harbor infrastructures: vessel tonnage continued to increase despite declining landing values. We have incorporated this result into both the Results and Discussion sections. These additions allow us to draw a stronger parallel between investments in vessels and investments in harbor works. Rather than responding directly to short-term fluctuations in the fishery, both forms of material investment appear to follow longer temporalities and development trajectories. We therefore revised the Discussion and Conclusion to emphasize that fishing capacity was reinforced through both maritime and terrestrial infrastructures, thereby extending the architectural argument beyond the shoreline. At the same time, while we recognize the value of approaching vessels as architectural objects, a more complete analysis of their design, construction, and material evolution would require a different body of evidence than that assembled in the present study.

# Reviewer report: Reviewer response for version 1

**DOI:** 10.21956/openreseurope.24982.r72266

**Published:** 2026-04-30

**Authors:** Jack Bouchard

**Affiliations:** 1 Rutgers University, New Brunswick, NJ, USA

This is an interesting piece which makes a small but useful contribution to our understand of historical fisheries. It is coming at the problem from the perspective of architecture, rather than fisheries studies, which offers a fresh perspective and the potential for more interdisciplinary thinking. I think that the question which the authors identify - what is the relationship between fishing, marine ecologies, and the built human coastal environment - is a worthwhile and provocative line of inquiry. (I am surprised, actually, that they do not invoke Westerdahl's idea of the Maritime Cultural Landscape - that seems to be, arguably, what the authors are interested in reconstructing). The last line - about treating coastal architecture not as passive but active -is well substantiated by the research. 

 In some ways, this is an interesting historical and anthropological problem which is limited by the scientific approach of the authors. The emphasis on quantitative methods rather than spending time with the archival material, especially from the French state's perspective, means that we only have a partial answer to the hypotheses posed at the beginning (as I think the authors acknowledge). I get the sense the authors did not quite find what they expected, but to their credit they use this to propose future lines of inquiry, and I think they have set up a set of conclusions which could be explored in other historical contexts. 

 The data generated by this study will nonetheless be useful to fisheries historians in the future. The author's conclusion that sardine landing declines does not have to do with environmental variability is a revealing and useful point, contributing to our global understanding of fisheries fluctuations in the past. 

 Although overall this is a good article, a few thoughts and suggestions. The article could be improved by a number of revisions, most of which serve to strengthen what the authors have already achieved.: 

 - The authors ought to be much clearer about the limitations (self-imposed) on the study. This isn't really a study of the Bay of Biscay, it is a study of one part of southern Brittany. In some places, it is more or less a study of one port. If they wish for southern Brittany to be a proxy for all of the Bay of Biscay then they will have to do more to make that case. Likewise, the focus on the sardine canning industry means that it would be hard to generalize the article's conclusions beyond the modern period. Unfortunately, these choices are not foregrounded in the study, and are not brought up much in discussing the conclusions. 

 - I think that the most interesting conclusion they develop is that infrastructure investment lags behind actual fluctuations in catches - that there is more intensive investment during periods of decline. (This tracks with Brian Payne's observations about state-backed advertising in the Canadian fishing industry, which intensified even as demand was plummeting). This opens some worthwhile lines of future research, and is a conclusion which can be tested in other settings around the Atlantic basin.

 This conclusion also suggests that fisheries historians should be paying more attention to politics - a point which the authors unfortunately don't lean into. But it would be worthwhile to reconstruct the decisions, lobbying, and debates which led to increasing French state investment in the sardine industry in the wake of declining catches. This suggests that rather than strictly focusing on coastal landscapes, we should be paying attention to archives of state power. 

 - At times, the argument becomes muddled across the article. The way the piece is structured it keeps coming back to conclusions, so that it feels a tad repetitive. But at times the authors have trouble committing to one or two clear arguments. Emblematic are some sentences at the start: "To a certain extent, we can argue that shore-based structures are a proxy measurement of fishing pressure, the latter increasing in relation to the growth of fish-processing facilities. In this paper, we explore the hypothesis that fluctuations in sardine landings influenced the ways we built on land. We discovered that this was not necessarily the case, since design and building practices relate to the agendas of a variety of agents other than fish, notably the state (in France through the *Ponts et Chaussées* technical offices). " These hedge and go back and forth, and dilute what are some useful observations. Clearer, more forceful argumentative language throughout would strengthen the piece.

If any, are all the source data and materials underlying the results available?

Yes

Is the work original in terms of material and argument?

Yes

Is the work clearly and cogently presented?

Partly

Is the argument persuasive and supported by evidence?

Yes

Does the research article contribute to the cultural, historical, social understanding of the field?

Yes

Does it sufficiently engage with relevant methodologies and secondary literature on the topic?

Yes

Reviewer Expertise:

Fisheries history, premodern (XV-XVII centuries); Marine environmental history

I confirm that I have read this submission and believe that I have an appropriate level of expertise to confirm that it is of an acceptable scientific standard, however I have significant reservations, as outlined above.

# Response

**Published:** 2026-06-25

**Authors:** Alice Nouvet

**Affiliations:** Universidade do Porto Faculdade de Arquitectura, Porto, Porto District, Portugal

We are grateful for the careful reading of the manuscript and the constructive comments. We greatly appreciate this assessment, which opens up further interdisciplinary lines of inquiry. In revising the manuscript, we have taken the remarks into account, focusing on three main points.

**Comment 1 :** “I am surprised, actually, that they do not invoke Westerdahl’s idea of Maritime Cultural Landscape – that seems to be, arguably what the authors are interested in reconstructing.”

**Response:** Regarding the suggested idea of engaging with Westerdahl’s concept of the Maritime Cultural Landscape, we agree that this framework is relevant to broader debates in maritime history. Our work shares with this perspective an interest in the relationships between maritime activity and the transformation of coastal environments. However, our study does not aim to reconstruct a maritime cultural landscape in the broader anthropological sense proposed by Westerdahl, encompassing navigation practices, toponymy, and the wider cultural dimensions of coastal communities. Rather, our focus is more specifically directed toward the material dimensions of coastal transformation, particularly the relationship between fluctuations in sardines’ population, fisheries shore-based infrastructure, and state-mediated investment in the built environment. In this sense, architecture is approached as a measurable and historically situated interface between marine exploitation and public policy. The goal is to incorporate biological marine histories—if not an animal perspective—within architectural history: hence the conscious effort to depart from terrestrial and anthropological approaches.

**Comment 2:** “The authors ought to be much clearer about the limitations (self-imposed) on the study. This isn’t really a study of the Bay of Biscay, it is a study of one part of southern Brittany. In some places, it is more or less a study of one port.”

**Response: **We appreciate this observation and have revised the manuscript to clarify the spatial scope of the study. The analysis operates across three spatial scales that reflect the availability and nature of the historical sources: the harbour of Douarnenez, South Brittany, and the broader French Atlantic façade. These scales are now presented more explicitly in the Methods and materials sections. We also clarify in the Conclusion that the objective is not to provide an exhaustive reconstruction of the Bay of Biscay as a whole, but rather to examine relationships between sardine landings, fishing capacity, and coastal investment through a set of interconnected case studies and datasets operating at different spatial scales.

**Comment 3:** “If they wish for southern Brittany to be a proxy for all of the Bay of Biscay then they will have to do more to make that case.”

**Response:** We agree that this point required clarification. South Brittany is not intended as a proxy for the entire Bay of Biscay. Rather, different spatial scales were examined using different datasets and methods. Detailed information on harbour infrastructures, fishing grounds, and vessel capacity was available for South Brittany and Douarnenez, whereas broader demographic and fisheries statistics were available at the scale of the French Atlantic façade. We have revised the Methods and materials sections to make this multi-scalar approach more explicit and to clarify the distinct role played by each dataset.

**Comment 4: **“(…) the focus on sardine canning industry means that it would be hard to generalize the article’s conclusions beyond the modern period.”

**Response:** We agree with this observation. The study is intentionally bounded by the temporal coverage of the available datasets, which span the period from the mid-nineteenth century to the mid-twentieth century. Our objective is not to generalize these conclusions beyond the modern period, but rather to use this historical case study to explore relationships between marine resources, fishing capacity, and coastal infrastructures.

**Comment 5** “This conclusion also suggests that fisheries historians should be paying more attention to politics – a point which the authors unfortunately don’t lean into. But it would be worthwhile to reconstruct the decisions, lobbying, and debates which led to increasing French state investment in the sardine industry in the wake of declining catches. This suggests that rather than strictly focusing on coastal landscapes, we should be paying attention to archives of state power.”

**Response:** We greatly appreciate this suggestion and agree that the political dimensions of fisheries management and public investment deserve further investigation. Indeed, the role of state institutions emerges repeatedly throughout our analysis, particularly through the activities of the Ponts et Chaussées. However, a detailed reconstruction of political decision-making, lobbying processes, and administrative debates falls beyond the scope of the present article. Our objective here was to assess the relationships between sardine fluctuations, fishing capacity, and shore-based infrastructures using a combination of spatial and quantitative evidence.

**Comment 6:** “At times, the argument becomes muddled across the article. The way the piece is structured it keeps coming back to conclusions, so that it feels a tad repetitive. But at times the authors have trouble committing to one or two clear arguments.”

**Response:** We appreciate this comment and have revised the manuscript accordingly. In the revised version, we have sought to make the central arguments more explicit and to reduce repetition across sections. In particular, we now structure the argument around three main relationships:

 (1) sardine behavior and the geography of fishing harbors,

 (2) the relationship between fishing grounds and landing sites, and

 (3) the relationship between sardine fluctuations and investments in fishing capacity and shore-based infrastructures.

 These themes are now articulated more consistently throughout the Results, Discussion, and Conclusion sections.
